# A longitudinal analysis of loneliness, social isolation and falls amongst older people in England

**DOI:** 10.1038/s41598-020-77104-z

**Published:** 2020-12-10

**Authors:** Feifei Bu, Jessica Abell, Paola Zaninotto, Daisy Fancourt

**Affiliations:** 1 Department of Behavioural Science and Health, University College London, 1-19 Torrington Place, London, WC1E 7HB UK; 2 Department of Epidemiology and Public Health, University College London, London, UK

**Keywords:** Psychology, Health care, Risk factors

## Abstract

Loneliness and social isolation have been identified as important predictors of various health outcomes, but little research has investigated their influence on falls. This study aimed to investigate the longitudinal association between loneliness, social isolation and falls amongst older adults in England, looking at both self-reported falls and falls that require hospital admissions. This study drew on large scale, nationally representative data from the English Longitudinal Study of Ageing linked with Hospital Episode Statistics. Data were analysed using survival analysis, with self-reported falls (total sample = 4013) and falls require hospital admission being modelled separately (total sample = 9285). There was a 5% increase in the hazard of self-reported falls relative to one point increase in loneliness independent of socio-demographic factors (HR: 1.05, 95% CI: 1.02–1.08), but the association was explained away by individual differences in health and life-style measures (HR: 1.03, 95% CI: 1.00–1.07). Both living alone (HR: 1.18, 95% CI: 1.07–1.32) and low social contact (HR: 1.04, 95% CI: 1.01–1.07) were associated with a greater hazard of self-reported falls even after controlling for socio-demographic, health and life-style differences. Similar results were also found for hospital admissions following a fall. Our findings were robust to a variety of model specifications.

## Introduction

Falls among older people are a major public health issue. It was estimated that 30–40% of older people fell at least once each year^1,2^, with over a third of falls resulting in injuries ranging from soft-tissue injury to hip fracture and intracranial trauma^3,4^. Consequently, falls are a leading cause of unintentional injuries and death^5^. Previous studies have reported that falls account for around 10% of visits to the emergency department and 6% of non-elective hospital admissions among older people^6^. Furthermore, falls have a detrimental impact on the functionality and mental health of older people^7,8^, and increase the risk of being admitted to care homes^9,10^. For these reasons, falls are a considerable financial burden to the health and social care system^11,12^. Estimates of their costs range from 0.85 to 1.5% of the total cost of health care in western countries^13^, equivalent to £981 million per year in the UK^14^.

Considerable research has been undertaken to investigate the causes and risk factors for falls among older people. Much of the work has focused on environment risks (e.g. loose carpet, slippery floor, unsuitable footwear, poor lighting) and biomedical factors such as muscle weakness, gait/balance deficit, dizziness, cognitive impairment, visual deficit, mobility limitation, poor nutritional status, medication, and depression^15–20^. There are also established links between falls and socio-demographic factors, such as age, gender, race and socioeconomic status^18,19^. However, little research work has been carried out to systematically investigate the relationship between falls and social factors. This is surprising given that social factors, in particular loneliness and social isolation, have long been recognised as important predictors of various health outcomes of older people, such as increased risk of all-cause mortality^21,22^, cardiovascular functioning^23,24^, cognitive impairment^25,26^, and depression^27,28^. Adapting from Cohen and Wills’^29^ theoretical model on health in general, social factors could influence the risk of falls through two mechanisms: (1) by providing material, instrumental and psychological resources, such as material aid, help with household tasks, and advice or guidance that reduce the risk of falls, and (2) by buffering the adverse effects of stressful events or other adverse psychological states such as depression that may predispose individuals to experience negative health outcomes such as falls.

Therefore, the aim of this study was to examine the relationship between loneliness, social isolation and the risk of falls among older people. Loneliness and social isolation are conceptually different in that social isolation measures objective social relationships, whereas loneliness (sometimes referred as perceived or subjective social isolation^30–32^) is a cognitive evaluation of the quantity and quality of one’s existing social relationships^33^. Following Cornwell and Waite’s^34^ suggestion, this study considered loneliness and social isolation as distinct concepts and explored if they were differentially associated with the risk of falls. We also examined different aspects of social isolation which might be related to the risk of falls through different mechanisms. Moreover, this study examined both self-reported falls (SR falls) and falls requiring hospital admission (HA falls) derived from administrative hospital records. This allowed us to explore whether loneliness and social isolation affected both the risk of fall itself and the activation of a specific clinical pathway involved in the treatment of a fall.

## Data and method

**Figure 1:**
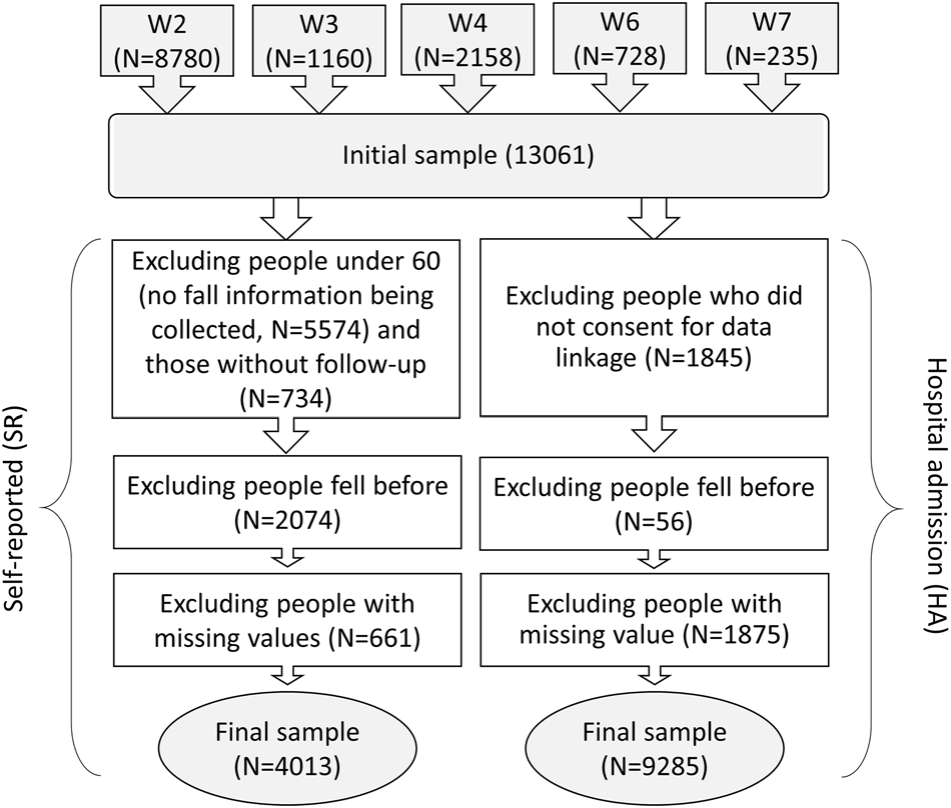
Sample selection diagram for the self-reported (SR) cohort and the hospital admission (HA) cohort.

The data came from the English Longitudinal Study of Ageing (ELSA). ELSA is a nationally representative longitudinal study of people aged 50 and over and their partners, living in private households in England. The original sample was drawn from participants who participated in the Health Survey in England (HSE) in 1998, 1999 and 2001^35^. The first wave of data collection commenced in 2002/2003, and participants have been followed biennially since. To maintain its representativeness, refreshment samples were added at wave 3, 4, 6 and 7. In this study, we restricted participants to core sample members, excluding partners under 50 years of age or partners who joined the household after the initial sampling. Further, we excluded participants who did not return self-completion questionnaires where our main variables of interest were measured (around 17% at wave 2). We used wave 2 (2004/5) as our baseline because some questions about isolation were not asked at wave 1. In addition to wave 2, we also included refreshments from later waves (see Fig. 1). In total, this provided us with an overall sample size of 13,061 participants.

### Falls

We derived fall incidents from two sources: self-reports from ELSA and administrative hospital records. In ELSA, participants aged 60 or over were asked at every wave (up to the latest available data from wave 8, 2016/2017) whether they have fallen down since their last interviews. Given the retrospective nature of this question, we used responses from subsequent waves to the baseline to identify people who had a fall in a later time. As no attempt was made to ask the date when they fell, the date of interview was used as the proxy of event time. In this study, we focused on the first fall after baseline, discarding subsequent falls. In the analysis of SR-falls, we restricted our sample to people aged 60 or over, as the questions about falls were not asked for younger cohorts. Further, we excluded individuals without any follow-up after their baseline interview, individuals who had reported falls at or before the baseline and those who had missing values in any of the predictors. This, as shown in Fig. 1, left us a sample of 4013 individuals for SR-falls.

In addition to whether an individual reported a fall, we also explored whether individuals were hospitalised as a result of a fall using linked Admitted Patient Care (APC) data from NHS Hospital Episode Statistics (HES). These data provided were available from February 1997 to January 2018, which allowed us to follow up ELSA participants from their baselines for a longer period of time, but also to retrieve hospital information prior to their baseline up to at least 5 years. Participants having a HA fall were identified as those who had any diagnosis code related to falls. Diagnoses in the APC data were coded according to the International Statistical Classification of Disease and Related Health Problems 10th Revision (ICD-10). Falls corresponded to the ICD-10 codes from W00 to W19. For time of event, we used the date of admittance following the fall. Participants who did not give consent for the data linkage (14% N = 1845) were excluded. Further, we also excluded a small number of sample members who had a fall within 5 years prior to their baseline interviews (N = 56) and those who have missing values in any of the predictors (see Fig. 1). This left us an analytical sample of 9285 individuals for HA-falls.

### Loneliness

Loneliness was measured using the three-item subscale from the revised UCLA loneliness scale^36^. The questions include: (1) how often do you feel lack companionship? (2) how often do you feel isolated from others? (3) how often do you feel left out? Responses to each question were scored on a three-point Likert scale ranging from hardly ever/never, to some of the time, to often. The resulting score is a loneliness index ranging from 3 to 9, with a higher value indicating greater levels of loneliness.

### Social isolation

We used two measures of social isolation: living alone and low social contact. Living alone was coded as a binary variable (alone vs. not alone). As one may expect, this variable was highly associated with marital status (Cramér's V = 0.84). However, there were around 22% of people without a spouse or partner who lived with someone else in the same household. Living alone was therefore considered a more appropriate measure for this study than marital status as it better captures the concept of social isolation.

Low of social contact was measured by the frequency of contacting relatives, children or friends. In the self-completion questionnaire, ELSA participants were asked how often they did the following activities with their children, including: (1) meet up (2) speak on the phone. The same set of questions was also asked about relatives and friends. These variables were recoded as binary variables indicating an activity happening at least weekly. Then a sum score was computed based on face-to-face and telephone contacts. The resulting index ranges from 0 to 6, with a higher value indicating a lower level of social contact.

### Covariates

Our analyses included socio-demographic variables, including age recoded into groups (50–59, 60–69, 70–79, 80+), gender (woman vs. man), ethnicity (white vs. non-white), and socio-economic status (using an index generated using principle component analysis based on education, social class and household wealth)^37^. In addition, we also included a set of health and life-style measures, such as self-reported long-standing illness, mobility, functional disability, vision, depression, and physical activity. Long-standing illness was coded as a binary variable indicating if participants had a long-standing illness limiting their daily activities. Mobility was calculated as a sum of the number of difficulties in ten activities, such as walking 100 yards, sitting for two hours, climbing stairs, lifting or carrying weights and so forth. The score ranged from 0 to 10. Functional disability was measured as sum scores of having difficulties in activities of daily living (ADL) and instrumental activities of daily living (IADL). The ADL score ranged from 0 to 6, and IADL from 0 to 9. Vision was derived from self-reported eyesight, which was recoded as a binary variable indicating having poor eyesight. Depression was measured using the eight-item Centre for Epidemiological Studies Depression (CES-D) scale. This is a validated tool that has been widely used across disciplines^38–41^. We removed the loneliness item from the CES-D scale to avoid double counting loneliness. The depression score was generated by adding responses to all seven items to provide a scale from 0 to 7 depressive symptoms. Physical activity was measured as a binary variable if participants took part in vigorous physical activity (e.g., running/jogging, swimming, cycling, gym workout, tennis, etc.) at least once a week.

### Statistical analysis

In this study, our event of interest was experiencing a fall. We used survival analysis to model the time from the baseline interview until a fall incident or the end of the follow-up period. In the analysis of self-reported falls (SR-falls), participants who had not had a fall were censored at the time of their last ELSA interviews, which varied across individuals. In the model of falls resulting in hospital admissions (HA-falls), participants were censored on 31 January 2018; the end of HES follow-up. Unlike the SR-falls model, participants in the HA cohort could also be censored due to death. Mortality was considered as a competing risk event to falls as it precluded the occurrence of falls. There are two main analytical approaches in the setting of competing risks: the Cox cause-specific hazards (CSH) model and the subdistribution hazards (SH) model^42–45^. Given there is no final consensus on which is a better approach, results from both the CSH and SH models are presented for the analysis of HA-falls.

Cox regression models built on the proportional hazards (PH) assumption, assuming the effect of a given covariate is constant across time^46^. This assumption was checked using both graphical diagnostics and statistical tests. There was no evidence the PH assumption was violated for the Cox models for SR falls, or the competing risks models for HA falls. All other model assumptions were also met.

As shown above in Fig. 1, both the SR and HA models were subject to missing data, resulting in a data reduction of 14% and 17%, respectively. So sensitivity analyses based on multiple imputation were performed to assess the influence of missing data on our findings. Multiple imputation by chained equations was implemented using the *mi impute chained* command to obtain 20 imputed data sets which corresponded to the percentage of incomplete cases^47^. As a further sensitivity analysis, we tested whether there was any moderating role of age or gender by including interaction terms. All analyses were carried out using Stata v15^48^.

### Ethics

Ethical approval for ELSA was granted from NHS Research Ethics Committees under the National Research and Ethics Service (NRES). All participants in our analyses provided informed consent for participation in the study and for linking their data with administrative health records. All methods in this study were performed in accordance with the relevant guidelines and regulations.

## Results

Table 1 shows the characteristics of the SR and HA cohorts. In the SR sample, nearly 52% of participants reported having fallen, which is much higher than the previously reported 28% using ELSA data^1^. This was because we assessed SR falls across multiple waves instead of in one wave. In the HA sample, fewer than 10% of participants had a fall-related hospital admission within the follow-up period. In general, the characteristics of these two samples were fairly similar, but the HA sample was younger (mean age of 64 vs. 69) as we did not have to restrict by age given HES data were available for all ages.

**Table 1:** Characteristics of the self-reported (SR) cohort and the hospital admission (HA) cohort at the baseline.

	SR cohort	HA cohort
Fall within follow-up period (%)	51.7	9.1
**Loneliness and isolation**		
Loneliness, mean (SD, range)	4.01 (1.44, 3–9)	4.16 (1.52, 3–9)
Living alone (%)	26.6	22.9
Low social contact, mean (SD, range)	2.88 (1.64, 0–6)	2.91 (1.64, 0–6)
**Socio-demographic**		
Woman (%)	50.8	53.6
**Age (%)**		
50–59	–	40.6
60–69	58.3	32.5
70–79	32.9	19.9
80+	8.8	7.0
Non-white (%)	2.5	2.2
Socioeconomic status index, mean (SD, range)	− 0.03 (1.35, − 2 to 2)	0.04 (1.35, − 2 to 2)
**Health and life-style**		
Limiting long-standing illness (%)	30.3	32.6
Mobility scale, mean (SD, range)	1.65 (2.21, 0–10)	1.80 (2.42, 0–10)
ADL scale, mean (SD, range)	0.27 (0.76, 0–6)	0.33 (0.89, 0–6)
IADL scale, mean (SD, range)	0.27(0.76, 0–9)	0.34 (0.87, 0–9)
Vigorous physical activities at least once a week (%)	29.7	30.1
Poor eye sight (%)	11.2	11.9
Depression score, mean (SD, range)	1.15 (1.57, 0–7)	1.34 (1.74, 0–7)
Observations (N)	4013	9285

### Self-reported falls

**Figure 2:**
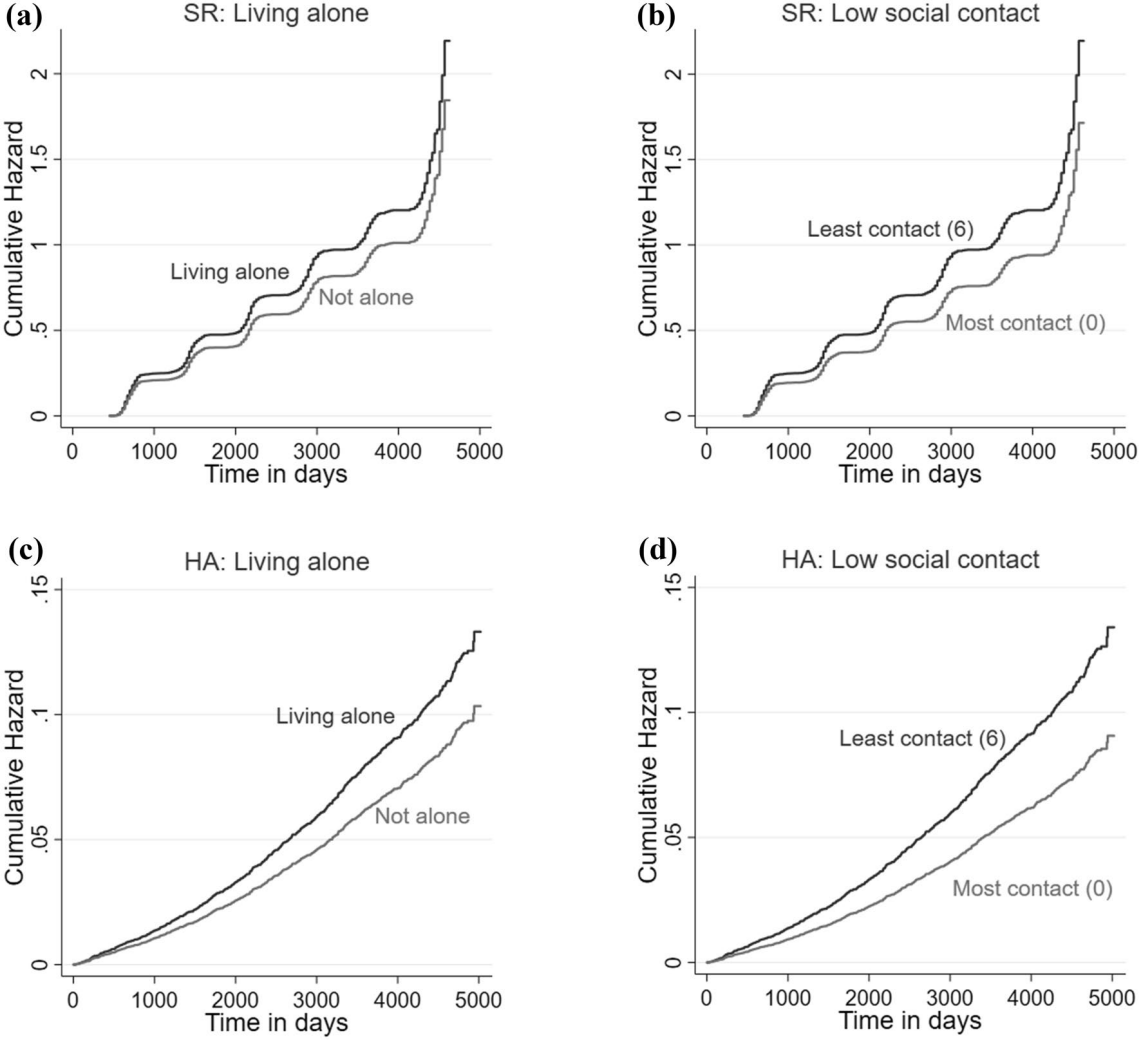
Estimated cumulative hazards by social isolation measures for the self-reported (SR) cohort and the hospital admission (HA) cohort.

When adjusting just for socio-demographic factors, there was a 5% increase in hazard of SR falls relative to one point increase in loneliness (HR: 1.05, 95% CI: 1.02–1.08), 17% for individuals who lived alone (HR: 1.17, 95% CI: 1.05–1.30) and 4% relative to one point increase in low social contact (HR: 1.04, 95% CI: 1.01–1.07) (Table 2). When additionally adjusting for health and life-style factors, the association for loneliness was attenuated, becoming insignificant, but the associations for living alone and low social contact remained, with individuals who lived alone having a 18% higher hazard of reporting a fall (HR: 1.18, 95% CI: 1.07–1.32), and individual with the least social contact (score of 6) having a hazard of 24% higher than those with the most social contact (score of 0) (HR: 1.04, 95% CI: 1.01–1.07). The estimated cumulative hazards by social isolation measures are presented in the upper panels of Fig. 2.

**Table 2:** Results from survival analysis models for self-reported (SR) and hospital admission (HA) cohorts.

SR falls HR [95% CI]		HA falls HR [95% CI]			
Model I	Model II	CSH model		SH model	
		Model I	Model II	Model I	Model II
**Loneliness**					
1.05**	1.03	1.08***	1.03	1.07**	1.03
[1.02–1.08]	[1.00–1.07]	[1.03–1.13]	[0.98–1.08]	[1.02–1.12]	[0.98–1.08]
**Living alone**					
1.17**	1.18**	1.25**	1.29***	1.20*	1.23*
[1.05–1.30]	[1.07–1.32]	[1.07–1.47]	[1.10–1.51]	[1.02–1.42]	[1.04–1.45]
**Low social contact**					
1.04**	1.04**	1.06**	1.07**	1.06*	1.06*
[1.01–1.07]	[1.01–1.07]	[1.02–1.11]	[1.02–1.11]	[1.01–1.10]	[1.01–1.10]
**N**					
4013	4013	9285	9285	9285	9285

Model I controlled for socio-demographic covariates; Model II controlled for socio-demographic, health and life-style covariates; *p < 0.05, **p < 0.01, ***p < 0.001; *HR *hazard ratio, *CI *confidence interval, *CSH* cause-specific hazards, *SH *subdistribution hazards.

### Falls resulting in hospital admissions

When adjusting just for socio-demographic covariates, loneliness, living alone and low social contact were all associated with a higher hazard of HA falls, both in the CSH and SH models (see Table 2). However, when adjusting for health and life-style related covariates, the association for loneliness was attenuated.

Older people living alone had a hazard rate which was 23–29% higher than those not living alone depending on the choice of statistical model (CHR: 1.29, 95% CI: 1.10–1.51; SHR: 1.23, 95% CI: 1.04–1.45), while individuals with the least social contact (score of 6) had a 36–42% higher hazard of reporting a fall than those with the most social contact (score of 0) (CHR: 1.07, 95% CI: 1.02–1.11; SHR: 1.06, 95% CI: 1.01–1.10). The estimated cumulative hazards from the CSH models by social isolation measures are presented in the lower panels of Fig. 2.

### Sensitivity analysis

When imputing missing data, there was no material difference in the results (Supplementary Table 1). When testing whether the effects of loneliness and social isolation differed by gender or age (Supplementary Tables 2 and 3), none of the interaction terms in the SR models was statistically significant. However, for HA falls, there was significant interaction effect between living alone and gender. The hazard ratio of living alone was 1.72 (95% CI: 1.34–2.19) for men but only 1.11 (95% CI: 0.93–1.34) for women.

## Discussion

This study explored the relationship between loneliness, social isolation and falls in older adults. Both social isolation measures, living alone and low social contact, were associated with a higher hazard of both self-reported falls and falls requiring hospital admittance amongst older adults, independent of individuals’ socio-demographic characteristics, as well as health and life-style factors. This builds on a previous cross-sectional study showing associations between loneliness, social isolation and falls, but extends them by providing longitudinal self-reported and objective hospital data over a follow-up period of up to 14 years^49^.

There are a number of mechanisms that could explain why social isolation are risk factors for falls. Co-residence and frequent social contacts with children, relatives and/or friends could help to identify and lessen the risks of falls. This could be related to environment risks, for example fixing a loose carpet or helping with housework. It might also concern biomedical risks, relating to health condition management. For instance, there is compelling evidence showing that social relationships increase access to health care and patient compliance with medications and therapies^50–53^, which may further contribute to reducing the risk of falls. However, it is also of note that we did not find persistent associations for loneliness after controlling for health and life-style factors, including long-standing illness, mobility, functional disability, vision, depression and physical activity. This pattern is consistent with findings from studies with shorter follow-up time that showed no relationship between loneliness and falls over a 1 year follow-up period^54,55^, and a previous study using ELSA data focusing on all-cause mortality which found the association between loneliness and mortality was not independent of demographic characteristics or health problems^22^. This suggests that it is objective social isolation rather than the subjective appraisal of one’s social relationships that is important. Whilst individuals who are less socially connected may engage less in physical activity, it is notable both that our results were found independent of exercise, mobility and functional disability, and also that living alone also had significant associations. One possible explanation is that social engagement with a spouse, other household members or people in the community increases an individual’s sense of purpose or alleviate stress^56,57^, which could then reduce their risk of falling. Indeed, purpose has been linked with factors such as better grip strength, lower levels of disability, and faster gait speed^58^. But this and other potential mechanisms will require further exploration.

Further, we found that both living alone and low social contact were also risk factors for hospital admittance following a fall. An important note here is that we do not know whether falls that required hospital admittance were more severe than the ones that did not, or whether individuals had to be admitted due to factors relating to their isolation. This is relevant in particular when considering the gender moderation we found for living alone: our findings suggest that living alone is a risk factor for hospital admittance following a fall for men, but not for women, but there is no gender moderation for having a fall. One potential explanation for this is that men living alone are more likely to be admitted to hospitals due to the lack of informal caring at home, compared with men who do not live alone. Indeed, it has previously been reported that men are more likely to rely on their spouses as a sole caregiver^59,60^. This is a key finding as it suggests that social isolation does not just affect the risk of falls, but also the risk of requiring hospital admittance following a fall. Given the cost of hospital admittance, if this association is driven by social factors rather than merely fall severity, this could have important health economic implications. Future studies into this distinction are thus encouraged.

A major strength of this study was the use of longitudinal research design to reduce the possibility of reverse causality. Moreover, through data linkage, we were able to investigate not only self-reported falls, which arguably are subject to recall bias or reporting bias, but also falls resulting in hospital admissions derived from hospital administrative records, allowing us to investigate potential distinctions and to cross-validate our findings. Our study, however, is not without limitations. First of all, we are aware a longitudinal design is not sufficient to establish causality. However, our findings were robust to different measurements of falls and to a range of statistical modelling and sensitivity analyses. As a second limitation, for the self-reported falls, we did not have information on the incident dates. Instead, interview dates were used as the time when falls were reported. We were aware that the ‘true’ date was bound to be earlier than the interview date and participants might have fallen more than once. However, the same principle was applied to all participants. Thus there was no reason to be believe this would lead to biased estimates in a systematic way. Moreover, this study examined only the first fall within the observational period. Future studies could look at the consistency of findings when exploring the relationship between social factors and multiple events. Finally, we explored only one aspect of treatment for falls: hospital admission using the APC data from HES. It remains unclear whether social isolation could increase engagement with other health care services following a fall. Consequently, future work would benefit from considering also medical treatment information from other data sources, in particular Accident and Emergency (A&E) data.

Falls are a common and often devastating problem for older people. In our study, over 50% of participants reported experiencing a fall within the study period. Approximately 9% of them had a hospital admission that was related to falls. Falls and consequent injuries are largely preventative, for example by environmental safety evaluations, behavioural instructions, exercise programs and so forth^2^. Our findings suggest the importance of additionally considering social isolation as a risk factor and exploring interventions that could help to reduce the risk amongst individuals who are socially isolated. Whilst this study uses data collected prior to the COVID-19 pandemic, its findings are particularly important in light of the pandemic as there is likely a heightened risk of loneliness and social isolation due to lockdown and social distancing measures. Future studies are encouraged to explore whether an increase in falls is a wider consequence of the pandemic and to consider interventions that could help to reduce this risk amongst older adults.

## Supplementary information

Supplementary information.
