# Supplementary material for: A longitudinal analysis of loneliness, social isolation and falls amongst older people in England
Source: Sci Rep. 2020 Dec 10;10:20064. doi: 10.1038/s41598-020-77104-z (PMC7730383; doi:10.1038/s41598-020-77104-z)
Supplement: Supplementary file 1 — Supplementary information. [file 41598_2020_77104_MOESM1_ESM.pdf]

Supplementary Tables

Table S1. Results Results from survival analysis models for self-reported (SR) and hospital admission (HA) cohorts

	SR falls HR [95% CI]			HA falls (CSH) HR [95% CI]		
	Model II-i	Model II-ii	Model II	Model II-i	Model II-ii	Model II
Loneliness	1.04*	1.05**	1.03	1.03	1.05	1.03
	[1.00—1.08]	[1.01—1.08]	[1.00—1.07]	[0.99-1.09]	[1.00-1.10]	[0.98—1.08]
Living alone	1.17**	--	1.18**	1.27**	--	1.29***
	[1.05—1.31]	--	[1.07—1.32]	[1.09-1.49]	--	[1.10—1.51]
Low social contact	--	1.04**	1.04**	--	1.06**	1.07**
	--	[1.01-1.07]	[1.01—1.07]	--	[1.02-1.11]	[1.02—1.11]
Observations (N)	4013	4013	4013	9285	9285	9285

Notes: All models controlled for socio-demographic, health and life-style covariates; * p<0.05, ** p<0.01, ***p<0.001; HR-hazard ratio, CI-confidence interval, CSH-cause-specific hazards, SH-subdistribution hazards

Table S2. Results from multiple imputation analyses for SR and HA falls

	SR falls	HA falls	
	Model II	Model II-CSH	Model II-SH
Loneliness	1.03	1.02	1.01
	[0.99—1.06]	[0.98—1.06]	[0.96—1.05]
Living alone	1.18**	1.30***	1.24**
	[1.07—1.30]	[1.13—1.50]	[1.07—1.43]
Low social contact	1.04**	1.06**	1.05**
	[1.01—1.07]	[1.01-1.10]	[1.01—1.10]
Observations (N)	4,692	11,160	11,160

Table S3. Results from models interacting loneliness and isolation with gender for SR (N=4,068) and HA falls (N=9,407)

	SR falls			HA falls (CSH)		
	Loneliness	Domestic isolation	Social isolation	Loneliness	Domestic isolation	Social isolation
Loneliness	1.04	1.03	1.03	1.00	1.02	1.03
	[0.99—1.10]	[0.99—1.06]	[1.00—1.07]	[0.92—1.08]	[0.98—1.08]	[0.98—1.08]
Living alone	1.19**	1.33**	1.18**	1.29**	1.73***	1.29***
	[1.06—1.32]	[1.13—1.58]	[1.06—1.32]	[1.10—1.51]	[1.35—2.19]	[1.10—1.51]
Low social contact	1.04**	1.04**	1.06**	1.07**	1.06**	1.06
	[1.01—1.07]	[1.01-1.07]	[1.02—1.10]	[1.02—1.11]	[1.02—1.11]	[0.99—1.13]
Woman	1.34*	1.30***	1.37**	0.96	1.35**	1.11
	[1.03—1.76]	[1.17—1.45]	[1.14—1.65]	[0.64—1.45]	[1.13—1.61]	[0.82—1.51]
Interaction term	0.98	0.84	0.97	1.05	0.64**	1.02
with woman	[0.92—1.04]	[0.68—1.03]	[0.92—1.02]	[0.96—1.14]	[0.48—0.87]	[0.93—1.11]

Notes: Fully adjusted model (Model II) with additional interaction terms; † $p<0.1$ * $p<0.05$, ** $p<0.01$, ***$p<0.001$

Table S4. Results from models interacting loneliness and isolation with age groups for SR (N=4,068) and HA falls (N=9,407)

	SR falls			HA falls (CSH)		
	Loneliness	Domestic isolation	Social isolation	Loneliness	Domestic isolation	Social isolation
Loneliness	1.04	1.03	1.03	1.02	1.03	1.03
	[1.00—1.09]	[1.00—1.07]	[1.00—1.07]	[0.92—1.12]	[0.98—1.08]	[0.98—1.08]
Living alone	1.25***	1.27**	1.24***	1.33***	1.51*	1.33***
	[1.12—1.39]	[1.11—1.48]	[1.11—1.38]	[1.14—1.55]	[1.03—2.20]	[1.14—1.55]
Low social contact	1.03*	1.03*	1.03	1.06*	1.06**	1.07
	[1.01—1.06]	[1.01-1.06]	[1.00—1.07]	[1.02—1.11]	[1.02—1.11]	[0.97—1.17]
Age 60-69	--	--	--	0.90	1.57***	1.45
	--	--	--	[0.51—1.61]	[1.24—1.98]	[0.94—2.24]
Age 70-79	1.55**	1.26***	1.25*	4.58***	4.03***	4.39***
	[1.17—2.05]	[1.13—1.41]	[1.03—1.51]	[2.64—7.85]	[3.20—5.07]	[2.93—6.59]
Age 80+	1.23	1.61***	1.53*	8.56***	7.35***	6.62***
	[0.77—1.96]	[1.25—2.08]	[1.09—2.16]	[4.55—16.09]	[5.33—10.12]	[4.06—10.77]
Interaction with	--	--	--	1.13	0.90	1.02
age 60-69	--	--	--	[1.00—1.28]	[0.55—1.46]	[0.90—1.15]
Interaction with	0.95	0.93	1.00	0.95	0.79	0.95
age 70-79	[0.89—1.01]	[0.75—1.15]	[0.94—1.06]	[0.85—1.08]	[0.51—1.21]	[0.84—1.07]
Interaction with	1.07	1.00	1.02	0.98	0.97	1.05
age 80+	[0.96—1.18]	[0.72—1.40]	[0.93—1.13]	[0.86—1.11]	[0.59—1.58]	[0.91—1.20]

Notes: Fully adjusted model (Model II) with additional interaction terms; † p<0.1 * p<0.05, ** p<0.01, ***p<0.001
